# Complete Microbial Fuel Cell Fabrication Using Additive Layer Manufacturing

**DOI:** 10.3390/molecules25133051

**Published:** 2020-07-03

**Authors:** Jiseon You, Hangbing Fan, Jonathan Winfield, Ioannis A. Ieropoulos

**Affiliations:** 1 Bristol BioEnergy Centre (BBiC), Bristol Robotics Laboratory, T Block, Frenchay Campus, University of the West of England, Bristol BS16 1QY, UK; jonathan.winfield@uwe.ac.uk; 2 Faculty of Engineering, University of Bristol, Bristol BS8 1TR, UK; hangbing.fan@manchester.ac.uk; 3 School of Mechanical, Aerospace and Civil Engineering, University of Manchester, Manchester M13 9PL, UK

**Keywords:** microbial fuel cell, additive manufacturing, 3D printing, PLA filament, carbon coating, membrane-less MFC, minimal surface-based structure

## Abstract

Improving the efficiency of microbial fuel cell (MFC) technology by enhancing the system performance and reducing the production cost is essential for commercialisation. In this study, building an additive manufacturing (AM)-built MFC comprising all 3D printed components such as anode, cathode and chassis was attempted for the first time. 3D printed base structures were made of low-cost, biodegradable polylactic acid (PLA) filaments. For both anode and cathode, two surface modification methods using either graphite or nickel powder were tested. The best performing anode material, carbon-coated non-conductive PLA filament, was comparable to the control modified carbon veil with a peak power of 376.7 µW (7.5 W m^−3^) in week 3. However, PLA-based AM cathodes underperformed regardless of the coating method, which limited the overall performance. The membrane-less design produced more stable and higher power output levels (520−570 µW, 7.4−8.1 W m^−3^) compared to the ceramic membrane control MFCs. As the final design, four AM-made membrane-less MFCs connected in series successfully powered a digital weather station, which shows the current status of low-cost 3D printed MFC development.

## 1. Introduction

For the commercialisation of the microbial fuel cell (MFC) technology, it is imperative to improve the efficiency by enhancing the system performance and reducing the production cost. This can be achieved through the optimisation of the system design for individual MFCs and stacks (in the case of scale-up), as well as the processes of manufacturing and assembly. Developing highly functional, low-cost and sustainable materials is also important. The manufacturing methods should precisely structure such materials in order to fabricate fully functional and efficient systems. In contrast to conventional manufacturing technologies such as subtractive manufacturing, which involves cutting away what is not needed from larger pieces of the material, additive manufacturing (AM) uses only the material needed, thus generating much less waste.

The AM process, also known as three-dimensional (3D) printing, builds three-dimensional structures from computer-aided design (CAD) models by adding material layer-by-layer. Technological progress has helped eliminate several limitations in manufacturing, and enabled the fabrication of products in complex geometry more precisely, with a shorter lead-time and minimum human intervention [1,2,3]. Consequently, applications of AM have been expanding rapidly in recent years, including industrial prototype printing, aerospace [4], medical implants [5,6] and the arts [7]. It also shows a great deal of potential in manufacturing novel designs of energy-generating technologies such as fuel cells, batteries, hydrogen, solar cells, carbon capture and storage, which were formerly unachievable through traditional manufacturing methods [8].

The most common application of AM for an MFC study is to build the chassis. Since AM enables the building of complex three-dimensional parts with a high degree of design freedom, novel MFC architectures were designed for specific applications [9,10,11,12]. 3D printed membranes have been tested, and some of them showed a comparable performance to cation exchange membranes [13,14,15]. Calignano et al. [16] demonstrated great potential for a 3D printed metal anode, using AlSi_10_Mg. Although metal 3D printing is currently very costly, it is worth considering for research purposes. On the other hand, much cheaper and biodegradable 3D filament material, such as polylactic acid (PLA)-based MFC anodes, have also been considered. High plasticity and easy access (entry-level 3D printers to print PLA filaments are available on the market) also make PLA attractive, although plain PLA filament is not electronically conductive. Our previous study [14] testing a commercially available conductive PLA filament (a combination of PLA and carbon black), showed that it is not suitable as the MFC anode due to its poor conductivity and relatively small surface area per unit volume. Therefore, further development for conductive 3D printable materials is needed for AM-built MFCs. An interesting approach to this was made by Bian et al. [17], who used non-conductive UV curable resin to print anode structures, and then modified the surface using copper electroless plating. Although copper is highly conductive (1.68 × 10^−6^ Ω∙cm), it is prone to corrosion in the MFC anode environment, unless external control of the anode potential is applied. The same group made an MFC carbonaceous anode using the same UV curable resin by a carbonisation process [18]. The anode showed a potential by producing a maximum power output of 233.5 ± 11.6 mW m^−2^, although the carbonisation process is usually a very energy-intensive process, which is less desirable. As mentioned previously, PLA has desirable properties as a 3D-printable MFC material, such as sound biocompatibility and biodegradability [19], as well as a low cost and high plasticity. Its low electrical conductivity could be overcome with help of adequate modifications.

On the other hand, not much information is available in the literature regarding 3D-printed MFC cathodes. Although Theodosiou et al. [15] developed 3D printable membrane electrode assemblies (MEA) by coating 3D printed membranes with carbon paint, the focus of the study was MEA, and therefore the cathode was not exhaustively or indeed separately investigated.

The present study therefore investigated low-cost but well-performing 3D printable materials for all MFC components, as well as improving the assembly process. The specific objectives of this study were: (1) to test 3D printable materials and surface modification methods for each component of MFC individually, i.e., anode, cathode and chassis; (2) to develop an MFC that is constructed entirely of components printed using AM technology; and (3) to demonstrate that AM-built MFCs are capable of powering an electrical device.

## 2. Results and Discussion

### 2.1. Anode Materials

The conductivity of the selected commercial conductive PLA was still poor compared to other conventional electrode materials such as carbon veil. It was therefore necessary to modify the electrode surface using a coating method. As can be seen in Table 1, carbon coating on either plain or conductive PLA increased conductivity significantly, even with a single layer of coating. The carbon loading increase of the double coating was almost four times higher than that of the single coating, which resulted in a great improvement in conductivity. Additional coating beyond the double coating started blocking holes of the electrode structure, thus decreasing the surface area, and there was no significant improvement in conductivity. Therefore, the double layer coating was adopted. On the other hand, the resistance of double nickel-coated samples was about 3 Ω (measuring distance of 1 cm, both parallel and perpendicular to the direction of printed layers), which was much lower than that of carbon-coated ones.

**Figure 1:**
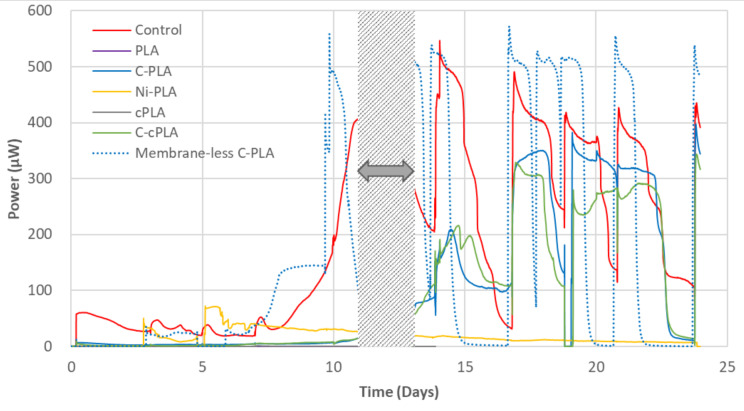
Power output of test anode materials. “C” and “c” in the sample names stand for “carbon-coated” and “conductive” respectively. For details of each test material, see Section 3.3. The grey-colored area indicates when data logging stopped for other electrochemical analysis of the cells. Data points are average values of triplicates.

Figure 1 shows power output produced from the tested MFCs with different anode materials for 24 days. All MFCs had the same control cathodes and terracotta membranes, and thus the difference in performance was a result of different anodes. The MFCs with unmodified PLA anodes generated no power during this period. Meanwhile, MFCs with carbon-coated anodes showed reproducible power outputs from week 3, which is typical behaviour of batch-fed MFCs with mature anodic biofilms. Although the control MFCs showed a faster maturation rate, the MFCs with C-PLA anodes achieved a comparable performance to the controls with a peak power of 376.7 µW (7.5 W m^−3^, normalised by the anodic volume) in week 3. In addition, the peak power of C-cPLA was lower than that of the C-PLA, indicating that the power generating performance is mainly attributed to the coating regardless of the base materials. The statistical analysis results also support these findings. The difference between C-cPLA and C-PLA was not significant, while there were significant differences (*p*-value less than 0.05) between the remaining test anodes, including membrane-less C-PLA for power output of each feeding cycle (*p*-value: < 0.001).

Initially, the power output of the MFCs with nickel-coated PLA (Ni-PLA) anode increased rapidly, and with a peak power of 70.3 µW (1.4 W m^−3^), outperformed other types of anode (about 3−5 µW) in week 1, due to its much higher surface conductivity. However, the power output then plateaued and began to decline slowly, becoming lower than MFCs with carbon-coated anodes in week 3. After 24 days of operation, it was observed that black particles had precipitated in the bottom of containers (Supplementary Materials, Figure S1), meaning that some of the nickel powder was detached from the coating. In the MFC anolyte environment, where the bacterial oxidation of organic matter takes place, this kind of nickel coating decomposition is thought to have detrimental effects on the anodic biofilms. Therefore, C-PLA was selected as the anode material for the final design.

### 2.2. Cathode Materials

**Figure 2:**
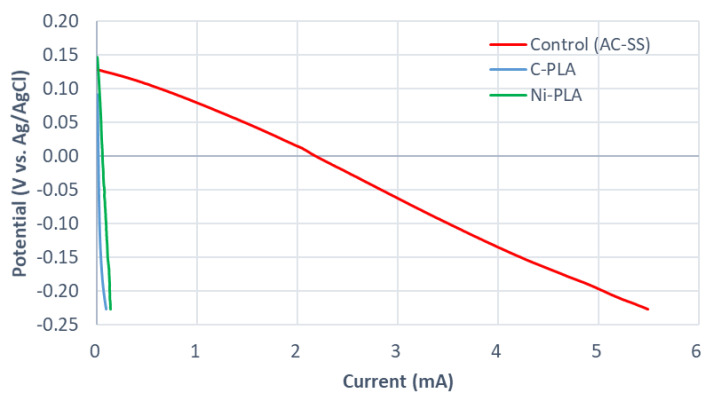
Linear sweep voltammetry (LSV) curves of the test cathode materials.

Figure 2 presents the linear sweep voltammetry (LSV) curves obtained for the comparison of the test cathode materials. Ni-PLA cathode showed the highest open circuit potential (OCP) of 174 mV (vs Ag/AgCl), followed by the control-activated carbon-stain steel (AC-SS) mesh cathode with OCP of 154 mV and C-PLA with the lowest OCP of 116 mV. However, both modified 3D-printed cathodes suffered significantly from high overpotentials, which resulted in underperformance compared to the control. Although at the same potential, Ni-PLA cathode showed a slightly higher current than C-PLA, C-PLA was chosen for the final design, in light of the anode material test.

In theory, most of the MFC anode materials can also be used as a cathode. However, the cathode, where the oxygen reduction reaction (ORR) takes place, often limits the overall performance [20]. In order to facilitate the ORR, many approaches have been suggested, including the use of mediators, highly conductive electrolytes, shorter electrode spacing and predominantly electrode modification with catalysts [21]. The results shown here indicate that developing new modification methods or 3D printable materials that provide higher conductivity and surface area is an essential prerequisite for AM-built MFCs.

### 2.3. Membrane-less 3D Printed MFCs

Although some of the 3D printable materials have shown promising results for MFC membranes [13,15], for design simplicity, membrane-less MFC configuration was also investigated. For this test, simply the ceramic membrane of the MFC with a C-PLA anode (described in Section 3.1.) was removed, and its performance compared to the same MFC with the membrane. The AC-SS cathode was used for both types of MFC. This test was carried out alongside the anode material test, and temporal power generation results are shown in Figure 1.

The membrane-less MFC showed faster maturation than the same electrode MFCs with a membrane, and achieved peak power of each feed ranging from 520−570 µW since day 10, while the ceramic membrane MFCs produced 320−400 µW after the maturation of the biofilm. However, in terms of power density normalised by the anodic volume, both types of MFC showed a similar performance, since the membrane-less MFC had a larger anode chamber volume (70 mL) than the ceramic membrane MFC (50 mL), due to absence of a membrane. Membrane-less MFCs usually have less internal resistance compared to those with membranes due to the absence of a membrane, although they suffer from low coulombic efficiency due to higher oxygen diffusion and substrate crossover [22,23]. This phenomenon was also observed in this study. The power output of membrane-less MFCs started to drop sharply 20 h after feeding, as the substrate was running out, which is almost twice as fast as MFCs with membranes. Therefore, more frequent feeding is required for batch-fed membrane-less MFCs to achieve a stable, high power-generating performance. When feeding the membrane-less MFCs once a day, they showed more stable power generation between day 17 and day 19. To better understand the energy benefit of MFC technology, the normalised energy recovery (NER) was suggested as an MFC performance metric [24,25]. Similar to this, energy generated from each feeding (20 mL of feedstock per feed) was calculated. In that case, MFCs with ceramic membranes generated 0.69 ± 0.20 kWh m^−3^, which is 21% higher than the energy generated from the membrane-less MFCs (0.57 ± 0.01 kWh m^−3^). Table 2 presents performance comparison results of the two test MFCs in different parameters. Despite the lower energy yield, for the final AM-built MFC design, the membrane-less type, as well as a more frequent feeding regime, was selected, due to the simpler design and higher power output.

### 2.4. Practical Application of the AM Built MFCs

The final stage of this study was set to realise an MFC design with all 3D printed components. Considering the overall MFC structure should be compact, the design of a single-chamber membrane-less MFC with an air cathode was determined.

**Figure 3:**
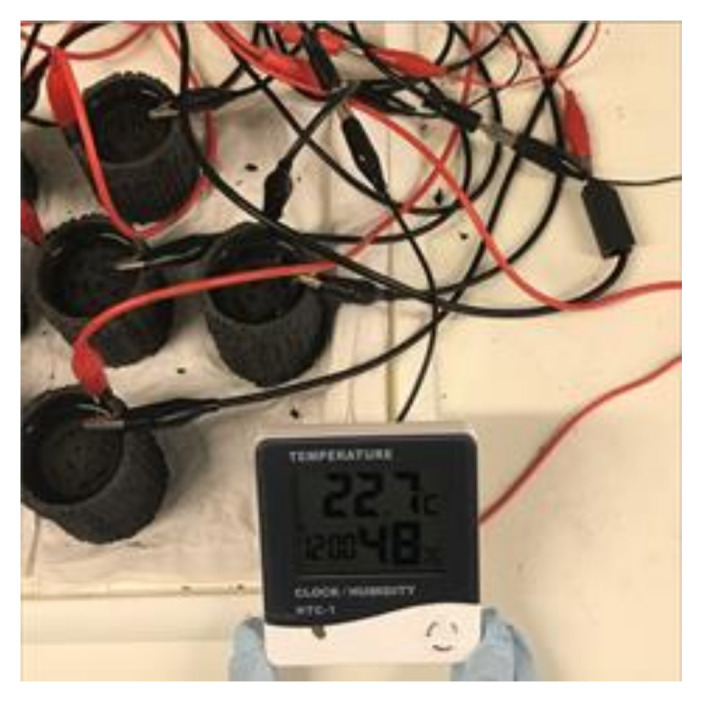
Demonstration of powering a digital weather station with additive manufacturing (AM)-built MFCs.

For demonstration purposes, it was attempted to power a low-power digital weather display device which normally requires one AAA (1.5 V) battery, using these AM-built MFCs. As shown in Figure 3, four AM-built MFCs connected in series were able to power the weather station successfully. To the best of the authors’ knowledge, this is the first time that complete AM-made MFCs have successfully powered an exemplar practical application, and this shows great promise for the further development of bioelectrochemical system technologies.

**Figure 4:**
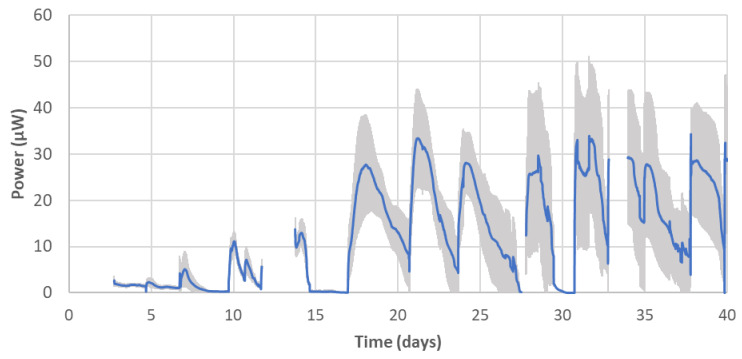
Power output of AM-built MFCs. Data points are average values of 16 MFCs. Sections where the graph line is discontinued indicate when voltage monitoring halted due to either electrochemical analysis or powering electrical devices, except the first three days of the open circuit period. Error bars stand for the standard deviation of the 16 MFCs.

The power-generating performance of the AM-built MFCs was monitored for 40 days (Figure 4). Compared to the non-AM MFCs (controls in Figure 1), AM-built MFCs showed a slower start-up, but similar to other partial-AM-built MFCs (test MFCs in Figure 1), they started producing a reproducible level of power output in each feed from week 3. After this time, the peak power of each feed ranged between 28 µW and 33 µW (0.7–0.8 W m^−3^), which is much less than both non-AM and partial-AM-built MFCs tested earlier in the study, although direct comparison is not reasonable due to the different designs and dimensions of the test MFCs. For the same period, these MFCs generated 0.07 ± 0.01 kWh m^−3^ from each feed. As discussed in the Section 2.2, one of the main causes of this low performance is thought to be the high overpotential of the 3D printed cathode, which had a limited ORR. Nevertheless, it is encouraging that complete AM-made MFCs operated as bioelectrochemical systems generating reproducible electrical power outputs.

## 3. Materials and Methods

### 3.1. Initial MFC Design

**Figure 5:**
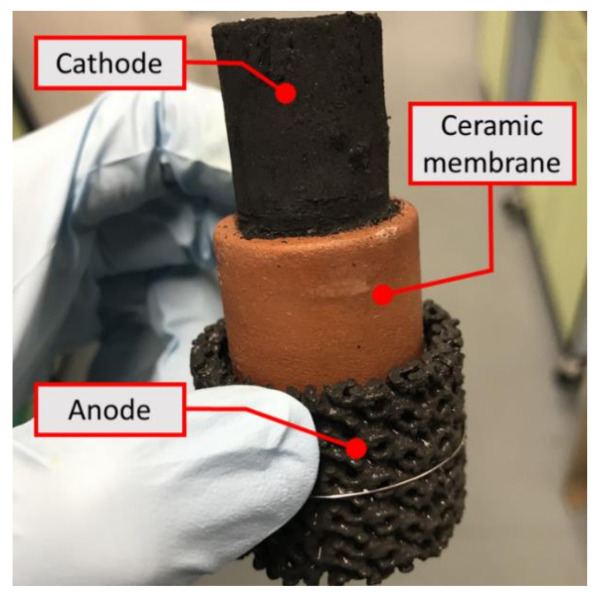
MFC configuration used in this study. A terracotta ceramic membrane was placed between the outer anode (3D printed anode in this photo) and the inner cathode.

The MFC design used in this study was based on cylindrical ceramic MFCs [26,27], with the ceramic membrane sandwiched between the external tubular anode and the internal cathode exposed to air (Figure 5). A top open-end terracotta cylinder (external diameter: 30 mm; thickness: 4 mm; height: 50 mm; Jain Scientific Suppliers, Ambala Cantt, India) was used as a membrane. The control cathode made of activated carbon pressed onto both sides of the AC-SS mesh backbone, as previously described [28], was placed inside the cylinder membrane, and its projected surface area was 43.4 cm^2^ (width: 62 mm; length: 70 mm; thickness: 2.5 mm). As for the control anode material, a piece of plain carbon veil (width: 90 mm; length: 300 mm; carbon loading: 20 g m^−2^; PRF Composite Materials, Poole, UK) was folded and wrapped around the ceramic membrane, resulting an outer surface area of 34.9 cm^2^ (external diameter: 37 mm; thickness: 3.5 mm; height: 30 mm). In order to fix the anode on the membrane, a nickel chrome wire (diameter: 0.45 mm; length: 20 mm) was used. Once assembled, they were placed in a 60 mL-volume cylindrical plastic container that acts as an anodic chamber, as well as an MFC chassis. Assembled MFC units can be found in  Supplementary Materials (Figure S2).

### 3.2. Fused Deposition Modelling (FDM)

**Figure 6:**
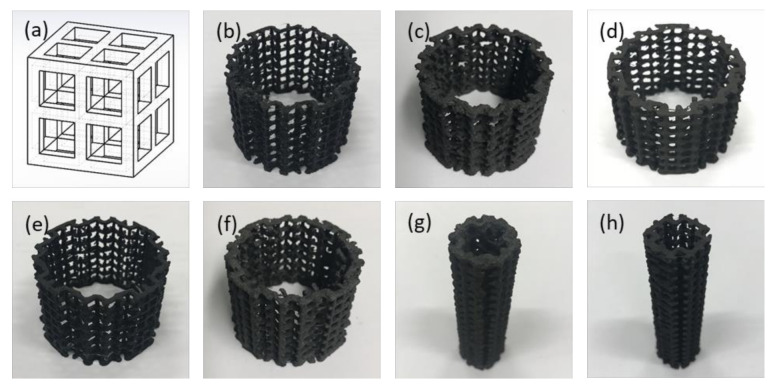
3D CAD design of the unit structure and test electrodes: (**a**) 3D CAD image of the electrode unit structure; (**b**) PLA anode; (**c**) C-PLA anode; (**d**) Ni-PLA anode; (**e**) cPLA anode; (**f**) C-cPLA anode; (**g**) C-PLA cathode; and (**h**) Ni-PLA cathode.

In order to test 3D printed electrodes, two types of PLA-based filaments with a diameter of 2.85 mm were selected. One is an electrically non-conductive PLA, and the other is an electrically conductive composite PLA (both purchased from the same manufacturer, Protoplant Inc., Vancouver, WA, USA). The conductive filament is a compound of PLA, a dispersant and carbon black (for conductivity). The 3D printed electrodes were designed through the repetition of a unit of simple cubic lattice structure, and manufactured by fused deposition modelling (FDM) using a bench top 3D printer (3D Touch, 3D Systems, Rock Hill, SC, USA) in the same size as the controls. The unit structure of 4 mm with a 1 mm strut (resolution: 0.25 mm) was selected (Figure 6a).

### 3.3. 3D Printed Electrode Surface Modification

In order to increase the surface conductivity of the 3D printed electrodes, electrodes were modified with additional carbon or nickel. For the carbon coating, carbon ink was prepared as previously described, using micronised natural graphite powder (3 g, Graphite Trading, Wolverhampton, UK), polyurethane rubber glue (2 g, Plasti Dip^®^, Plasti Dip International, Blaine, MN, USA) and petroleum spirit (10 mL, BDH Limited, Brighouse, UK) [29]. The other surface modification of nickel coating was performed using a spray paint containing nickel powder (Nickel Screening Compound Plus-NSCP, Electrolube, Ashby-de-la-Zouch, UK), which is an electrically conductive coating material based on nickel powder in a thermoplastic resin. It was then simply sprayed onto the 3D printed electrodes to cover the entire surface, then air-dried at least for one day. Nickel was chosen due to its low resistivity (6.99 × 10^−6^ Ω∙cm) and sound corrosion resistance.

The electrical resistance of the tested 3D printed parts was measured using a digital multimeter (Fluke 289, Fluke Inc., Everett, WA, USA). Two probes of the multimeter were placed 1 cm apart, both parallel and perpendicular to the direction of layers of the 3D printed parts, and resistance between the two points was measured.

In total, five different materials were selected for the anode material test (non-conductive PLA; carbon-coated non-conductive PLA; nickel-coated non-conductive PLA; conductive PLA; and carbon-coated conductive PLA), with plain carbon veil as a control. For the cathode material test, graphite carbon-coated and nickel-coated non-conductive PLA cathodes were selected and compared with the control AC-SS cathode described in Section 3.1. The details of the test electrode materials are listed in Table 3, and test electrodes are shown in Figure 6.

### 3.4. Final MFC Design for Practical Demonstration

**Figure 7:**
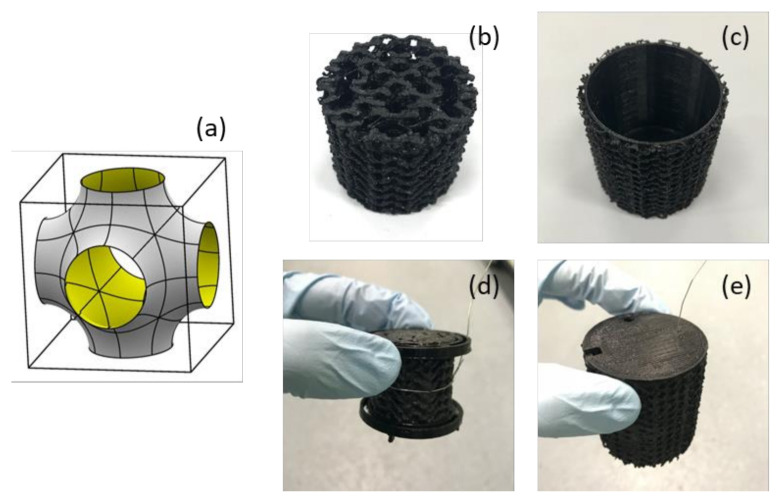
Final design of AM-built MFC: (**a**) Schwarz P surface unit cell design; (**b**) 3D printed anode; (**c**) 3D printed cathode; (**d**) anode with holders; and (**e**) whole assembly.

Based on the findings from previous tests (results presented in Section 2.1, Section 2.2 and Section 2.3), a new design of 3D printed MFC was built for the final demonstration (Figure 7). Since the cathode was a major limiting factor, a larger surface area for the cathode compared to the anode was sought. The AM-built cylindrical MFC is a membrane-less design with an internal anode and external cathode that also works as a chassis. A cylindrical anode (diameter: 30 mm; height: 30 mm; resolution: 0.25 mm), which had two 3 mm-thick gaps inside to achieve a better internal coating result, was placed inside the cathode (external diameter: 47 mm; thickness: 2 mm; height: 50 mm; resolution: 0.25 mm). For increasing surface area of both electrodes, a Schwarz P surface-based structure was chosen as a unit cell structure (Figure 7a), since this surface possesses a high surface-to-volume ratio and porosity [30,31]. The surface areas of both anode and cathode calculated by a 3D CAD software (Rhino 6, Robert McNeel & Associates, Seattle, WA, USA) were 167.3 cm^2^ and 349.0 cm^2^, respectively. C-PLA was used to build both anode and cathode electrodes. These two electrodes were fixed by two ring-shaped holders printed in plain PLA, in order to avoid a short circuit. These holders keep a fixed inter-electrode distance of 5 mm between the two electrodes. Moreover, both holders were designed with a 2 mm gap for the liquid flow-through, and to ensure that the wire connected to the anode does not come into contact with the cathode (Figure 7d). A circular lid was printed in plain PLA with three holes for substrate feeding and electrode connections. This also helps reduce anolyte evaporation loss. A total anolyte volume of 40 mL was kept throughout the test. For a demonstration purpose, on day 12, it was attempted to power a digital weather station (HTC-1, Sinotimer, Wenzhou, China), using electricity produced by the AM-built MFCs.

### 3.5. MFC Operation and Data Recording

Each MFC was inoculated with 50 mL of sewage sludge (Wessex Water, Bath, UK), enriched with 1% tryptone and 0.5% yeast extract, and then operated for two days until the open circuit voltage stabilised. Subsequently, 20 mL of feedstock (0.1% tryptone, 0.05% yeast extract and 0.2% sodium acetate in distilled water) was fed to each MFC after removing 10–15 mL of anolyte every two days. MFCs were left open circuit for the first two days, and then external resistors (initially 1 kΩ for the controls and 3 kΩ for the other test sets) were connected. As the internal resistance of the MFCs decreased, the value of external resistance was adjusted to match the internal one for maximizing power output [14,32]. During the experiments, the power output of all MFCs was recorded in real time in volts every five minutes, using a PicoLog 1216 Data Logger (Pico Technology Ltd., St Neots, UK). All tests were performed in triplicates in a temperature-controlled environment, at 22 ± 2 °C.

### 3.6. Electrochemical Analysis

LSV was used to analyse the cathode materials on day 11, using SP-50 Potentiostat (Bio-Logic Science Instruments, Seyssinet-Pariset, France) at a scan rate of 0.25 mV sec^−1^. The subjected cathodes and the carbon veil anode were connected as working and counter electrodes, respectively, and the curves in which the cathodic potential varies with current were obtained [33]. Between each LSV run, there was one h break for anode biofilm to recover.

### 3.7. Statistical Analysis

To evaluate if the differences between test anodes and membrane-less type in terms of power generation performance are statistically meaningful, an analysis of variance (ordinary one-way ANOVA and unpaired t-test) was performed using GraphPad Prism 8 (GraphPad Software, San Diego, CA, USA). Peak powers produced from four feeding cycles (five cycles for the membrane-less type) between days 16 and 24 were calculated. This resulted in 63 data points for five anode types (C-PLA; Ni-PLA; C-cPLA; membrane-less C-PLA; and control).

## 4. Conclusions

In this study, building an MFC consisting of all 3D printed components such as anode, cathode and chassis was attempted for the first time. Simple and cost-effective carbon coating, even on a non-conductive PLA base structure, showed comparable performance with a conventional carbon veil anode. However, the 3D printed cathode with the same modification underperformed in comparison to the control activated carbon air cathode, which limited the overall performance. The final design of the AM-made MFCs, with all parts 3D printed, demonstrated that the manufacturing of monolithic MFCs, which avoids complex manufacturing and assembly, is indeed feasible.

This study also revealed areas of further research. The development of new modification methods or highly conductive 3D printable materials is essential. The design optimisation of each component, as well as a whole MFC unit, could be investigated further, since rapid prototyping is one of the biggest merits of AM technology. Tests for the long-term performance of AM-built MFCs should be carried out if a prolonged operation is expected in its application.

AM can be a great tool for the commercialisation of the MFC technology by expediting the optimisation procedure of bespoke designs, as well as new materials for the whole system. It is advantageous in markets with a high demand for customisation, flexibility, design complexity and precision. AM can also reduce or even eliminate the need for assembly (i.e., monolithic design), which could allow for cost-effective, enhanced and consistent performance with minimum human error. With novel materials and designs, AM will therefore be able to drive the commercialisation of MFC technology.

## Figures and Tables

**Table 1:** The measured resistance and carbon loading increase of carbon-coated samples.

Samples	Resistance-Parallel to the Direction of Layers ^1)^ (Ω)	Resistance-Perpendicular to the Direction of Layers ^1)^ (Ω)	Carbon Increase (mg cm^−2^)
PLA	n/m ^2)^	n/m	n/a ^3)^
Single coated C-PLA ^4)^	1100	2025	3.0
Double coated C-PLA	240	370	12.5
cPLA ^5)^	1235	1955	n/a
Single coated C-cPLA	385	540	2.6
Double coated C-cPLA	175	305	12.4

^1)^ measuring distance: 1 cm, ^2)^ n/m: not measurable, ^3)^ n/a: not applicable, ^4)^ “C” stands for “carbon-coated”, ^5)^ cPLA refers to the commercial conductive polylactic acid (PLA) selected for this study.

**Table 2:** Performance comparison between membrane-less and ceramic membrane microbial fuel cells (MFCs).

Membrane Type	Peak Power of Each Feed		Energy Generation from Each Feed	
	Absolute power (µW)	Power to volume ^1)^ (W m^−3^)	Energy (mWh)	Energy to volume ^2)^ (kWh m^−3^)
Membrane-less	520–570	7.4–8.1	11.38 ± 0.20	0.57 ± 0.01
Ceramic membrane	320–400	6.4–8.0	13.83 ± 0.41	0.69 ± 0.20

^1)^ anodic chamber volume, ^2)^ feedstock volume added per feed.

**Table 3:** Details of 3D printed electrodes tested in the study.

Sample Name	PLA	C-PLA	Ni-PLA	cPLA	C-cPLA
Base filament material	Non-conductive PLA			Conductive PLA	
Surface modification	n/a	Carbon coating	Nickel coating	n/a	Carbon coating
Tested as	Anode	Anode, cathode	Anode, cathode	Anode	Anode
Dimension	(Anode) external diameter: 41 mm, height: 30 mm, thickness: 3.5 mm) (Cathode) external diameter: 20 mm, height: 70 mm, thickness: 4.0 mm)				
Surface area ^1)^	(Anode) 158.5 cm^2^ (Cathode) 96.2 cm^2^				

^1)^ Calculated values using a 3D computer-aided design (CAD) software (Rhino 6).

## Supplementary Materials

The following are available online at https://www.mdpi.com/1420-3049/25/13/3051/s1, Figure S1: Photos of anolyte precipitates from a nickel coated anode after two weeks of running: (left) top view, (right) side view of the container after removing the MFC components; and Figure S2: Assembled MFCs for anode material test: (left) MFC unit assembly and (right) experimental set up.

## References

[1] Wong K.V., Hernandez A. (2012). A Review of Additive Manufacturing. ISRN Mech. Eng..

[2] Yuan L., Ding S., Wen C. (2019). Additive manufacturing technology for porous metal implant applications and triple minimal surface structures: A review. Bioact. Mater..

[3] Tai X.Y., Zhakeyev A., Wang H., Jiao K., Zhang H., Xuan J. (2019). Accelerating Fuel Cell Development with Additive Manufacturing Technologies: State of the Art, Opportunities and Challenges. Fuel Cells.

[4] Griffiths L. (2015). 3D printing for Aerospace: “Additive manufacturing will change the game forever”. TCT Magazine.

[5] Murphy S.V., Atala A. (2014). 3D bioprinting of tissues and organs. Nat. Biotechnol..

[6] Rasperini G., Pilipchuk S.P., Flanagan C.L., Park C.H., Pagni G., Hollister S.J., Giannobile W.V. (2015). 3D-printed bioresorbable scaffold for periodontal repair. J. Dent. Res..

[7] Walters P., Davies K. (2010). 3D printing for artists: Research and creative practice. Rapp. J. Nor. Print Assoc..

[8] Zhakeyev A., Wang P., Zhang L., Shu W., Wang H., Xuan J. (2017). Additive Manufacturing: Unlocking the Evolution of Energy Materials. Adv. Sci..

[9] Walters P., Ieropoulos I., McGoran D. Digital fabrication of a novel bio-actuator for bio-robotic art and design. Proceedings of the International Conference on Digital Printing Technologies.

[10] Papaharalabos G., Greenman J., Melhuish C., Ieropoulos I. (2015). A novel small scale Microbial Fuel Cell design for increased electricity generation and waste water treatment. Int. J. Hydro. Energy.

[11] Massaglia G., Gerosa M., Agostino V., Cingolani A., Sacco A., Saracco G., Margaria V., Quaglio M. (2017). Fluid Dynamic Modeling for Microbial Fuel Cell Based Biosensor Optimization. Fuel Cells.

[12] Agostino V., Massaglia G., Gerosa M., Sacco A., Saracco G., Margaria V., Quaglio M. (2020). Environmental electroactive consortia as reusable biosensing element for freshwater toxicity monitoring. New Biotechnol..

[13] Philamore H., Rossiter J., Walters P., Winfield J., Ieropoulos I. (2015). Cast and 3D printed ion exchange membranes for monolithic microbial fuel cell fabrication. J. Power Sources.

[14] You J., Preen R.J., Bull L., Greenman J., Ieropoulos I. (2017). 3D printed components of microbial fuel cells: Towards monolithic microbial fuel cell fabrication using additive layer manufacturing. Sustain Energy Techn..

[15] Theodosiou P., Greenman J., Ieropoulos I. (2019). Towards monolithically printed Mfcs: Development of a 3d-printable membrane electrode assembly (mea). Int. J. Hydro. Energy.

[16] Calignano F., Tommasi T., Manfredi D., Chiolerio A. (2015). Additive Manufacturing of a Microbial Fuel Cell—A detailed study. Sci. Rep..

[17] Bian B., Wang C., Hu M., Yang Z., Cai X., Shi D., Yang J. (2018). Application of 3D Printed Porous Copper Anode in Microbial Fuel Cells. Front. Energy Res..

[18] Bian B., Shi D., Cai X., Hu M., Guo Q., Zhang C., Wang Q., Sun A.X., Yang J. (2018). 3D printed porous carbon anode for enhanced power generation in microbial fuel cell. Nano Energy.

[19] Shive M.S., Anderson J.M. (1997). Biodegradation and biocompatibility of PLA and PLGA microspheres. Adv. Drug Deliv. Rev..

[20] Santoro C., Arbizzani C., Erable B., Ieropoulos I. (2017). Microbial fuel cells: From fundamentals to applications. A review. J. Power Sources.

[21] Rismani-Yazdi H., Carver S.M., Christy A.D., Tuovinen O.H. (2008). Cathodic limitations in microbial fuel cells: An overview. J. Power Sources.

[22] Du Z., Li Q., Tong M., Li S., Li H. (2008). Electricity Generation Using Membrane-less Microbial Fuel Cell during Wastewater Treatment. Chin. J. Chem. Eng..

[23] Leong J.X., Daud W.R.W., Ghasemi M., Ben Liew K., Ismail M. (2013). Ion exchange membranes as separators in microbial fuel cells for bioenergy conversion: A comprehensive review. Renew. Sustain. Energy Rev..

[24] He Z. (2012). Microbial Fuel Cells: Now Let us Talk about Energy. Environ. Sci. Technol..

[25] He Z. (2017). Development of Microbial Fuel Cells Needs To Go beyond “Power Density”. ACS Energy Lett..

[26] Preen R.J., You J., Bull L., Ieropoulos I. (2018). Design mining microbial fuel cell cascades. Soft Comput..

[27] Gajda I., Greenman J., Santoro C., Serov A., Atanassov P., Melhuish C., Ieropoulos I. (2018). Multi-functional microbial fuel cells for power, treatment and electro-osmotic purification of urine. J. Chem. Technol. Biotechnol..

[28] Walter X.A., Greenman J., Ieropoulos I. (2018). Binder materials for the cathodes applied to self-stratifying membraneless microbial fuel cell. Bioelectrochemistry.

[29] Winfield J., Chambers L.D., Stinchcombe A., Rossiter J., Ieropoulos I. (2014). The power of glove: Soft microbial fuel cell for low-power electronics. J. Power Sources.

[30] Schwarz H.A. (1890). Gesammelte Mathematische Abhandlungen.

[31] Shin J., Kim S., Jeong D., Lee H.G., Lee D., Lim J.Y., Kim J. (2012). Finite Element Analysis of Schwarz P Surface Pore Geometries for Tissue-Engineered Scaffolds. Math. Probl. Eng..

[32] Song Y.E., Boghani H., Lee T., Premier G., Kim H.S., Kim B.G., Jeon B.-H., Kim J.R. (2016). Maximum Power Point Tracking to Increase the Power Production and Treatment Efficiency of a Continuously Operated Flat-Plate Microbial Fuel Cell. Energy Technol..

[33] Nahir T.M., Clark R.A., Bowden E.F. (1994). Linear-Sweep Voltammetry of Irreversible Electron Transfer in Surface-Confined Species Using the Marcus Theory. Anal. Chem..

