# Supplementary material for: Complete Microbial Fuel Cell Fabrication Using Additive Layer Manufacturing
Source: Molecules. 2020 Jul 3;25(13):3051. doi: 10.3390/molecules25133051 (PMC7412530; doi:10.3390/molecules25133051)
Supplement: Supplementary file 1 [file molecules-25-03051-s001.pdf]

molecules

MDPI

Supplementary Materials

Complete Microbial Fuel Cell Fabrication Using Additive Layer Manufacturing

Jiseon You [1,*], Hangbing Fan [2,3], Jonathan Winfield [1] and Ioannis A. Ieropoulos [1,*]

1 Bristol BioEnergy Centre (BBiC), Bristol Robotics Laboratory, T Block, Frenchay Campus, University of the West of England, Bristol, BS16 1QY, UK; jonathan.winfield@uwe.ac.uk
2 Faculty of Engineering, University of Bristol, Bristol, BS8 1TR, UK; hangbing.fan@manchester.ac.uk
3 School of Mechanical, Aerospace and Civil Engineering, University of Manchester, Manchester, M13 9PL, UK
* Correspondence: jiseon.you@uwe.ac.uk (J.Y.), ioannis.ieropoulos@brl.ac.uk (I.A.I.); Tel.: +44-1173286318 (I.A.I.)

Academic Editor: María José Salar-Garcia
Received: 27 May 2020; Accepted: 30 June 2020; Published: date

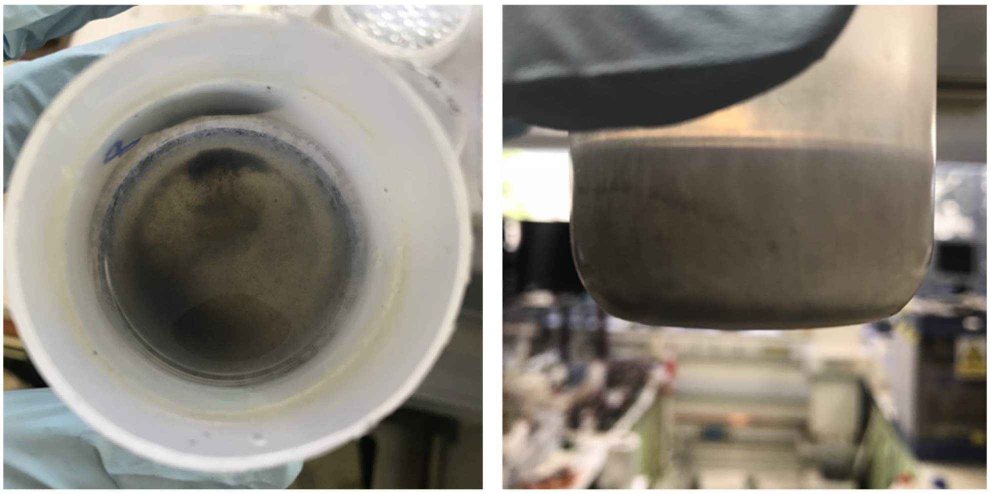

Figure S1. Photos of anolyte precipitates from a nickel coated anode after 2 weeks of running: (left) top view, (right) side view of the container after removing the MFC components

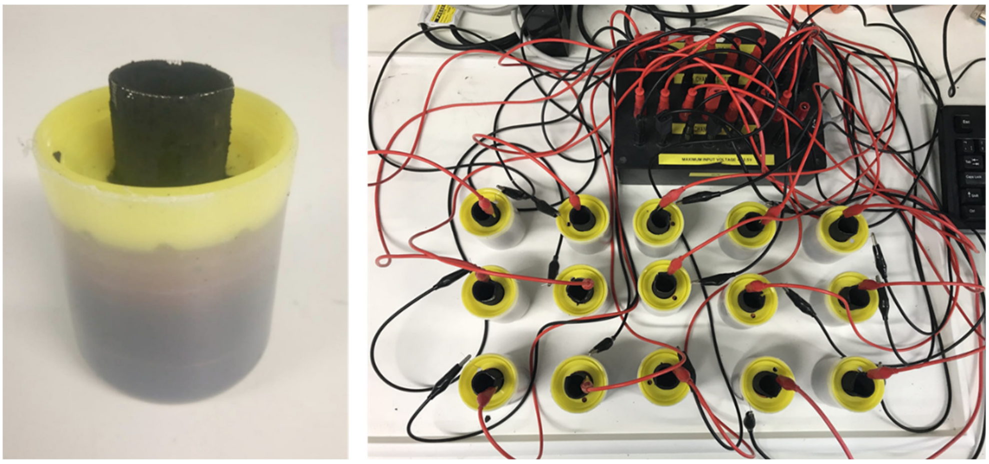

Figure S2. Assembled MFCs for anode material test: (left) MFC unit assembly, (right) experimental set up
